# Reduced Susceptibility to Chlorhexidine among *Staphylococcus aureus* Isolates in Israel: Phenotypic and Genotypic Tolerance

**DOI:** 10.3390/antibiotics10030342

**Published:** 2021-03-23

**Authors:** Maya Azrad, Chen Shmuel, Tamar Leshem, Zohar Hamo, Moti Baum, Assaf Rokney, Keren Agay-Shay, Avi Peretz

**Affiliations:** 1 Clinical Microbiology Laboratory, The Baruch Padeh Medical Center, Poriya, Tiberias 1528001, Israel; mazrad@poria.health.gov.il (M.A.); goldameir2000@gmail.com (C.S.); tleshem@poria.health.gov.il (T.L.); Zhamo@poria.health.gov.il (Z.H.); 2 Central Government Laboratories, Israel Ministry of Health, Jerusalem 9446724, Israel; moti.baum@moh.gov.il (M.B.); assaf.rokney@moh.gov.il (A.R.); 3 The Azrieli Faculty of Medicine, Bar-Ilan University, Safed 1311502, Israel; keren.agay-shay@biu.ac.il

**Keywords:** chlorhexidine, reduced susceptibility, healthcare-associated infections, *S. aureus*, genotypic and phenotypic susceptibility

## Abstract

Antiseptic use for body decolonization is the main activity applied to prevent healthcare-associated infections, including those caused by *S. aureus*. Consequentially, tolerance to several antiseptics such as chlorhexidine gluconate (CHG) has developed. This study aimed to estimate the prevalence of CHG tolerance among *S. aureus* strains in Israel and to evaluate factors that may affect this tolerance. Furthermore, it tested the associations between phenotypic and genotypic CHG tolerance. *S. aureus* strains (*n* = 190) were isolated from clinical samples of patients admitted to various medical institutions in Israel. Phenotypic susceptibility to CHG was assessed by determining minimum inhibitory concentration (MIC) and minimum bactericidal concentration (MBC). Genotypic tolerance was detected using real-time PCR for detection of *qac* A/B genes. MIC for the antibiotic mupirocin was determined using the Etest method. Presence of the Panton–Valentine Leucocidin (*pvl*) toxin, *mec*A and *mec*C genes was detected using an eazyplex^®^ MRSAplus kit (AmplexDiagnostics GmbH, Gars, Germany). CHG tolerance was observed in 13.15% of the isolates. An association between phenotypic and genotypic tolerance to CHG was observed. Phenotypic tolerance to CHG was associated with methicillin resistance but not with mupirocin resistance. Additionally, most of the CHG-tolerant strains were isolated from blood cultures. In conclusion, this work shed light on the prevalence of reduced susceptibility to CHG among *S. aureus* strains in Israel and on the characteristics of tolerant strains. CHG-tolerant strains were more common than methicillin-resistant ones in samples from invasive infections. Further research should be performed to evaluate risk factors for the development of CHG tolerance.

## 1. Introduction

*Staphylococcus aureus* (*S. aureus*) is one of the most common causes of human diseases, ranging from skin and soft tissue infections to pneumonia, meningitis, and sepsis [1]. *S. aureus* infections and, specifically, methicillin-resistant *S. aureus* (MRSA) infections are frequent both in the community and in healthcare institutions (nosocomial infections), are associated with high morbidity and mortality rates, and incur high medical costs [2]. Magill et al. reported that *S. aureus* is the second most common cause of healthcare-associated infections in the United States [3]. It was estimated that 40–60% of all nosocomial *S. aureus* infections are due to MRSA [4].

One of the main preventive activities against healthcare-associated infections is the use of antiseptics and biocides for hand and body decolonization [5] in order to reduce the bacterial load of pathogens from the colonized or infected body [6]. One example of such an antiseptic agent is chlorhexidine gluconate (CHG). Chlorhexidine (1,6-bis(4-chlorophenylbiguanido)hexane) is active against a wide range of fungi, some viruses [7,8,9,10], and gram-positive and gram-negative bacteria, especially MRSA and vancomycin-resistant enterococci [11]. It is extensively utilized because of its long-lasting bactericidal effect and its satisfactory tolerability and safety profiles [6].

However, the massive use of CHG has led to the development of reduced susceptibility to CHG among bacteria in general and in *S. aureus* specifically (reviewed in [6,12]). CHG antiseptic tolerance is mediated by efflux pumps that are encoded by several genes, such as *qac* A, *qac* B, and *smr* [6,13]. Interestingly, strains that show phenotypic tolerance to CHG do not always carry tolerance genes and vice versa. One of the most important findings regarding these genes is their association with resistance to antimicrobial agents [6]. This phenomenon is explained by either cross-resistance or co-resistance. Cross-resistance means that one efflux pump can remove both biocides and antibiotics. Co-resistance occurs when tolerance genes and antibiotic resistance genes are colocalized on the same mobile genetic element [14].

The prevalence of reduced susceptibility to CHG among *S. aureus* strains in Israel is unknown. This study aimed to assess its prevalence and to search for factors that may be associated with CHG tolerance. Furthermore, it assessed the possible association between phenotypic tolerance and *qac* A/B presence. We found that 13.15% of our strains were CHG-tolerant; phenotypic tolerance was associated with genotypic tolerance to CHG. Some bacterial characteristics were associated with CHG tolerance.

## 2. Results

### 2.1. Characteristics of Clinical S. aureus Isolates

This study included 92 MRSA and 98 methicillin-sensitive *S. aureus* (MSSA) isolates recovered from blood cultures (35.3% isolates) and wound cultures (64.7% isolates) (Table 1). Thirty-nine (20.5%) isolates carried the gene for the *pvl* toxin; most (28/39, 71.8%) of these *pvl*-positive isolates were MRSA ones (*p* = 0.001). All MRSA isolates contained the *mec*A gene. None of the isolates carried the *mec*C gene (data not shown).

**Figure 1:**
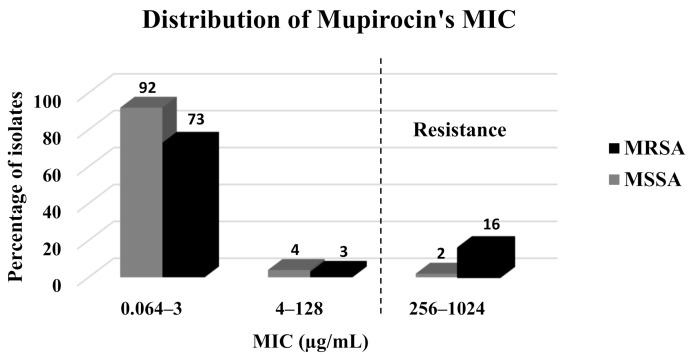
Distribution of mupirocin’s MIC (mg/L) among the clinical isolates.

Mupirocin resistance was observed in 9.5% of the isolates and was significantly more prevalent among the MRSA isolates (16/92, 17.4%) as compared to the MSSA isolates (2/98, 2.05%) (*p* < 0.001) (Table 1, Figure 1). In addition, the mean and median MICs were significantly higher among the MRSA isolates (15.3 mg/L and 0.19 mg/L, respectively) as compared to the MSSA isolates (178.75 mg/L and 0.125 mg/L, respectively) (*p* = 0.023) (Table 1).

### 2.2. Susceptibility to CHG among Clinical S. aureus Isolates

**Figure 2:**
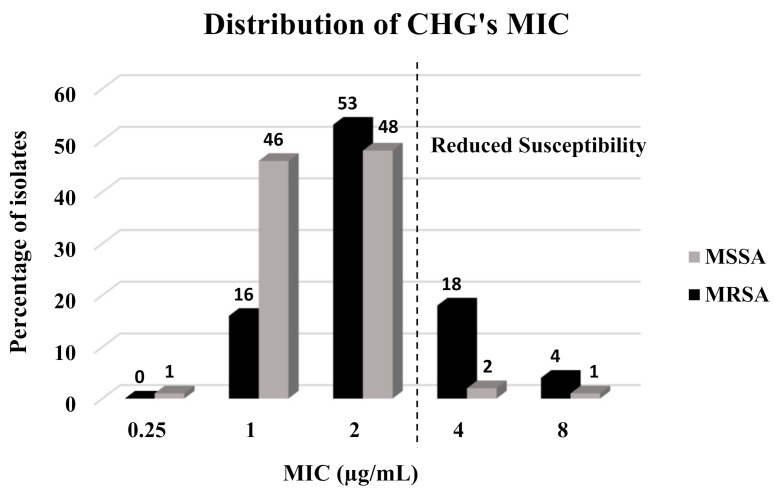
Distribution of CHG’s MICs (mg/L) among clinical isolates.

The prevalence of reduced susceptibility to CHG was 13.15% (25/190) (Table 2), with most of the tolerant isolates belonging to the MRSA group (22/25, 88%) (*p* < 0.001) (Table 2, Figure 2). Mean CHG’s MICs were higher and the MIC distribution was significantly different between MRSA and MSSA isolates (2.5 mg/L and 1.62 mg/L, respectively) (*p* < 0.001). Mean CHG MBCs were also higher and the distribution was significantly different between MRSA and MSSA isolates (2.45 mg/L and 2.23 mg/L, respectively) (*p* < 0.001). In parallel, out of the 25 (13.15%) isolates that carried the *qac* A/B gene, 21 (84%) were MRSA isolates and only four (16%) were MSSA isolates (*p* < 0.001).

### 2.3. The Associations between Phenotypic and Genotypic Tolerance to CHG

An association was found between the genotypic and phenotypic tolerance to CHG (*p* < 0.001) (Table 3). When only MSSA isolates were assessed, no association between the genotype and the phenotype was found (*p* = 0.717). In contrast, in MRSA strains, the phenotype and the genotype of CHG susceptibility were linked (*p* = 0.004) (Table 4).

### 2.4. The Associations between Reduced Susceptibility to CHG and Methicillin Resistance

The prevalence rate of reduced susceptibility to CHG among MRSA isolates was 8-fold higher than the prevalence rate among MSSA isolates (24% of the MRSA isolates were CHG-tolerant as compared to 3% of the MSSA isolates) (*p <* 0.0001) (Table 3).

### 2.5. The Associations between Reduced Susceptibility to CHG and Mupirocin Resistance

Since CHG is used for body decolonization, a possible association between CHG tolerance and resistance to mupirocin, which is an antibiotic that is used superficially, was considered. Most (61%) mupirocin-resistant isolates were susceptible to CHG. The rate of reduced CHG susceptibility was significantly lower among mupirocin-sensitive isolates as compared to mupirocin-resistant isolates (10.5% vs. 39%, respectively, *p* = 0.001) (Table 3). It should be noted that the average mupirocin MIC was higher among isolates with a reduced CHG susceptibility (257.44 mg/L) as compared to susceptible isolates (69.74 mg/L). However, the difference in the distribution of mupirocin MIC was not statistically significant (*p* = 0.066).

There was no association between CHG tolerance and mupirocin resistance among MRSA isolates. In addition, no significant difference was seen in the distribution of mupirocin MIC for CHG-tolerant vs. CHG-susceptible MRSA isolates (Table 4). In contrast, among the MSSA isolates, while 50% of the mupirocin-resistant isolates were CHG tolerant, only 2.1% of the mupirocin-sensitive isolates were tolerant (*p* = 0.000) (Table 4). However, the difference in the distribution of the mupirocin’s MIC was not statistically significant between the CHG-tolerant isolates as compared to the CHG-susceptible MSSA isolates (*p* = 0.846).

### 2.6. The Association between Reduced Susceptibility to CHG and the pvl Toxin Presence

No association was found between reduced susceptibility to CHG and the *pvl* toxin presence (*p* = 0.548, Table 3) neither in MRSA nor in MSSA isolates (Table 4).

### 2.7. The Association between Reduced Susceptibility to CHG and the Sample Source

As mentioned above, 67 isolates were originally isolated from blood cultures and 123 isolates were isolated from wounds. The highest percentage of CHG-tolerant isolates was found among isolates from blood cultures (21%). An association between the sample source and the tolerance to CHG was identified (*p* = 0.020, Table 3). However, no such association was found when analyzing only the MSSA isolates (Table 4). Among the MRSA isolates, there was an association between the sample source and CHG tolerance; reduced susceptibility to CHG was found in 39.4% and 15.3% of the blood culture isolates and the wound isolates, respectively (*p* = 0.009) (Table 4).

## 3. Discussion

The rates of phenotypic tolerance to CHG vary between countries, ranging from 0.06% to 35% [15,16,17,18]. In addition, the median CHG’s MIC values differ in different countries [15,16,17,18]. Similarly, the rate of genotypic tolerance differs between different countries (reviewed in [6]). There are several possible reasons for these differences, including different infection control and intervention programs and different protocols for CHG use. In the current study performed on clinical isolates collected in Israel, 13.15% of the clinical *S. aureus* strains exhibited reduced susceptibility to CHG. In parallel, out of 190 isolates, 13.15% harbored the *qac* A/B genes.

Although some *qac* A/B-negative strains were phenotypically tolerant to CHG and some *qac* A/B-positive strains were phenotypically CHG-susceptible, an association was found between phenotypic and genotypic tolerance to CHG, particularly among the MRSA strains. It is not surprising to find CHG-tolerant *S. aureus* isolates that lack the *qac* A/B genes, since there are over 11 genes encoding the efflux pumps that mediate tolerance to biocides [6]; the current work tested for the presence of only two. On the other hand, the presence of these genes does not necessarily provide phenotypic tolerance as was seen with antibiotic resistance genes. This may be due to carriage of the gene without its expression. Various studies have reported on this discrepancy between phenotypic and genotypic tolerance [16,17,18,19]. It should be noted that none of the previous studies found a statistically significant association between genotypic and phenotypic susceptibility to CHG.

To identify risk factors that may be associated with CHG tolerance, we first tested resistance to methicillin. The prevalence of CHG tolerance and the average CHG’s MIC were higher among MRSA strains as compared to MSSA strains. These findings align with previous reports, in which MRSA strains were less susceptible to CHG as compared to MSSA strains [20,21,22]. These observations are concerning in light of the common use of CHG as a disinfectant, particularly for MRSA infections. On the one hand, the clinical significance of the high CHG’s MIC among MRSA isolates remains unknown, as CHG is used at much higher concentrations than the tested MICs [23]. On the other hand, there is some evidence of its clinical impact; for example, one study reported on the spread of an MRSA strain with an elevated MIC following the use of a standard CHG disinfection protocol in an intensive care unit (ICU) [24]. In addition, there are subpopulations of staphylococci with heterogeneous tolerance to CHG [6]. Therefore, the phenomenon of reduced susceptibility of *S. aureus*, specifically of MRSA strains, to CHG should be further investigated.

As with the phenotypic tolerance, the prevalence of genotypic tolerance was also higher among the MRSA strains (22.8%) versus the MSSA strains (4.1%). The reported prevalence of *qac* A/B genes in MRSA isolates varies across different geographic areas, from 0.9% in the USA [25] to 83.3% in Malaysia [26] (reviewed in [12]). MSSA isolates show a *qac* A/B prevalence of 2–12% [27,28,29]. As already mentioned, these differences may result from diverse decolonization and prevention policies and different protocols for CHG use, among other factors.

The mupirocin resistance rate was 9.5% among all isolates, 2.05% among MSSA isolates, and 17.4% among MRSA isolates. Most previous studies analyzed mupirocin resistance rates among MRSA samples only; these rates ranged between 0% and 65% (reviewed in [7,30]). This broad range may result from the diverse mupirocin treatment protocols for MRSA infections (including different frequencies of mupirocin use), different study populations, different study settings (community vs. hospital, clinical isolates vs. colonizing isolates), among other factors. A recent systematic review reported on a higher prevalence of mupirocin resistance among MRSA isolates as compared to MSSA isolates (13.8% and 7.6%, respectively) [31] as shown in our work. A possible explanation for this finding is that mupirocin is more frequently used against MRSA infections than for MSSA infections.

Regarding the association between mupirocin resistance and CHG tolerance, we surprisingly found an opposite association, i.e., most (72%) of the CHG-tolerant strains were susceptible to mupirocin. One possible explanation is that mupirocin and CHG are not administered simultaneously. Thus, the treated strain develops resistance/tolerance either to mupirocin or to CHG, but not to both. While no reports of assessment of this association were found in the literature, one study did report on coexistence of a low level of resistance to mupirocin in MRSA with *qac* A/B genes, which was associated with elevated risk for persistent carriage following decolonization [32]. Thus, even though no association was found between CHG tolerance and mupirocin resistance in the current study, it is important to continue monitoring *S. aureus* isolates and their susceptibility to mupirocin and CHG.

The presence of the *pvl* toxin was the third tested characteristic. The *pvl* toxin creates pores in leukocyte membranes and is associated with increased bacterial virulence. In the current work, 20.5% of the strains carried the *pvl* toxin gene. A comparison with other studies concluded that *pvl* prevalence among *S. aureus* isolates varies between geographical areas or the type of patients (e.g., children vs. adults) [33], from 3% to 75% [34,35,36]. Interestingly, the majority of *pvl*-positive strains in our study were MRSA, an observation that aligns with earlier reports [33,37,38,39]. For example, a study performed in Alaska in 2000 reported that while no MSSA isolates carried the *pvl* genes, 92% of MRSA isolates had the toxin [39]. However, several studies found the opposite pattern, where the *pvl* toxin was more common among MSSA strains [40,41,42]. These discrepancies may derive from different characteristics of study cohorts, including patient type and age, sample sources, and sample size. Although we hypothesized that *pvl* presence may be linked to CHG tolerance, no such association was found. No previous studies investigated the relationships between *pvl* presence and CHG tolerance. Thus, further research should be performed on this aspect.

The last characteristic that we tested was the sample source and its possible association with CHG tolerance. The CHG tolerance rate was higher among the strains isolated from blood cultures as compared to wounds. This association was also seen among the MRSA strains. To the best of our knowledge, this is the first evidence of an association between phenotypic CHG tolerance in *S. aureus* and the invasiveness of the condition. These observations strengthen two previous reports that analyzed *S. aureus* strains isolated from children and showed that CHG’s genotypic tolerance was associated with a higher rate of invasive infection and with central venous catheters [43,44]. This is likely related to the fact that invasive *S. aureus* strains have the ability to form biofilms that are impermeable to antibiotics and biocides. Therefore, it is reasonable that strains causing invasive infections such as bacteremia are more tolerant to antiseptics.

The study had several limitations; first, no data regarding patient exposure to CHG were collected; second, other data regarding risk factors for CHG tolerance, such as patients’ underlying diseases, previous hospital admissions, presence of central venous lines, etc., should have been collected.

In summary, this work shed light on the prevalence of reduced CHG susceptibility among *S. aureus* strains in Israel and on the characteristics of tolerated strains. It can be concluded that CHG-tolerant strains are more often isolated from invasive infections and are more likely methicillin-resistant. Additionally, phenotypic tolerance to CHG is associated with the presence of the *qac* A/B genes. This is the first time that such a study was conducted in Israel. Further research should be performed to evaluate risk factors for the development of CHG tolerance.

## 4. Materials and Methods

### 4.1. Bacterial Strains

The study included 190 *S. aureus* isolates (98 MSSA and 92 MRSA isolates) from human blood and wound cultures. Ninety-two strains (52 MSSA and 40 MRSA strains) were isolated from blood and wound cultures of patients (age range: 0 to 100 years) admitted to the Padeh Poriya Medical Centre between January 2018 and December 2019. These isolates were recovered as part of the routine diagnosis workup at the clinical microbiology laboratory. Briefly, colonies suspected to be *S. aureus*-positive were isolated on blood agar (BD Diagnostics, Sparks, MD, USA) and on a selective chromogenic growth medium Chromagar^TM^ MRSA/MSSA (Hy Laboratories Ltd., Rehovot, Israel), and then incubated at 37 ± 1 °C for 18–24 h. Confirmatory tests, including gram staining, a catalase test, and a rapid agglutination test, for simultaneous detection of the fibrinogen affinity antigen (clumping factor), protein A, and capsular polysaccharides (Pastorex™ Staph Plus, BIO RAD, Marnes-la-Coquette, France) were performed on each isolate. Final identification of *S. aureus* was performed using the Bruker Biotyper system (Bruker Daltonics, Bremen, Germany), which is based on the matrix-assisted laser desorption ionization–time of flight (MALDI–TOF) technique. Methicillin resistance testing was performed according to the routine laboratory protocol, by testing the isolate susceptibility to cefoxitin.

The other 98 strains (46 MSSA and 52 MRSA strains) were randomly collected at the *S. aureus* National Reference Centre, Israel Ministry of Health, Jerusalem, Israel. These isolates were originally isolated from wound and blood cultures of patients (age range: 0 to 100 years) admitted to various medical institutions in Israel between May 2015 and February 2018.

### 4.2. Antibiotic Susceptibility Testing (AST)

Susceptibility to mupirocin was assessed using the Etest method that is used to determine the MIC, i.e., the minimal concentration (mg/L) of a given antibiotic that inhibits growth of a particular bacterium under specific experimental conditions. All isolates were grown on blood agar (Hy Laboratories Ltd., Rehovot, Israel) at 37 ± 1 °C for 18–24 h. Several colonies were then suspended in saline to a turbidity of 0.5 McFarland. The suspensions were seeded on Mueller–Hinton agar plates (Hy Laboratories Ltd., Rehovot, Israel), after which an Etest strip of mupirocin (bioMérieux, Durham, NC, United States) was added to each agar plate. All plates were incubated at 37 ± 1 °C for 18–24 h. Mupirocin susceptibility was determined in accordance with the European Committee on Antimicrobial Susceptibility Testing (EUCAST) 2018 guidelines [45].

### 4.3. Phenotypic Susceptibility to CHG

#### 4.3.1. Broth Microdilution for MIC Determination

**Figure 3:**
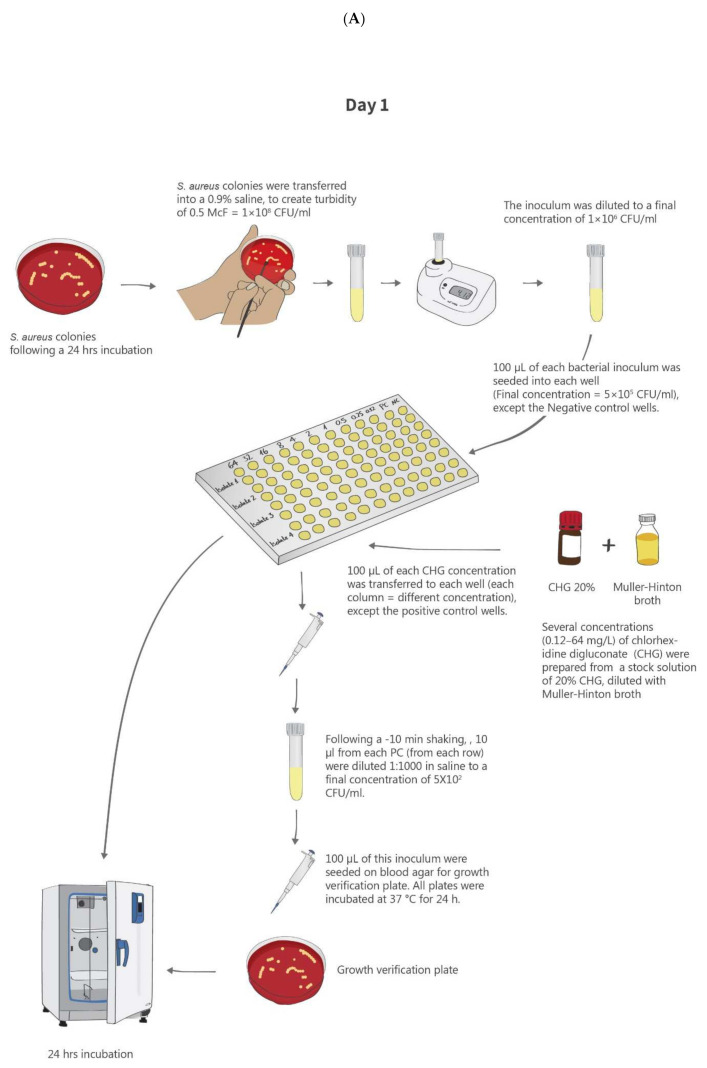

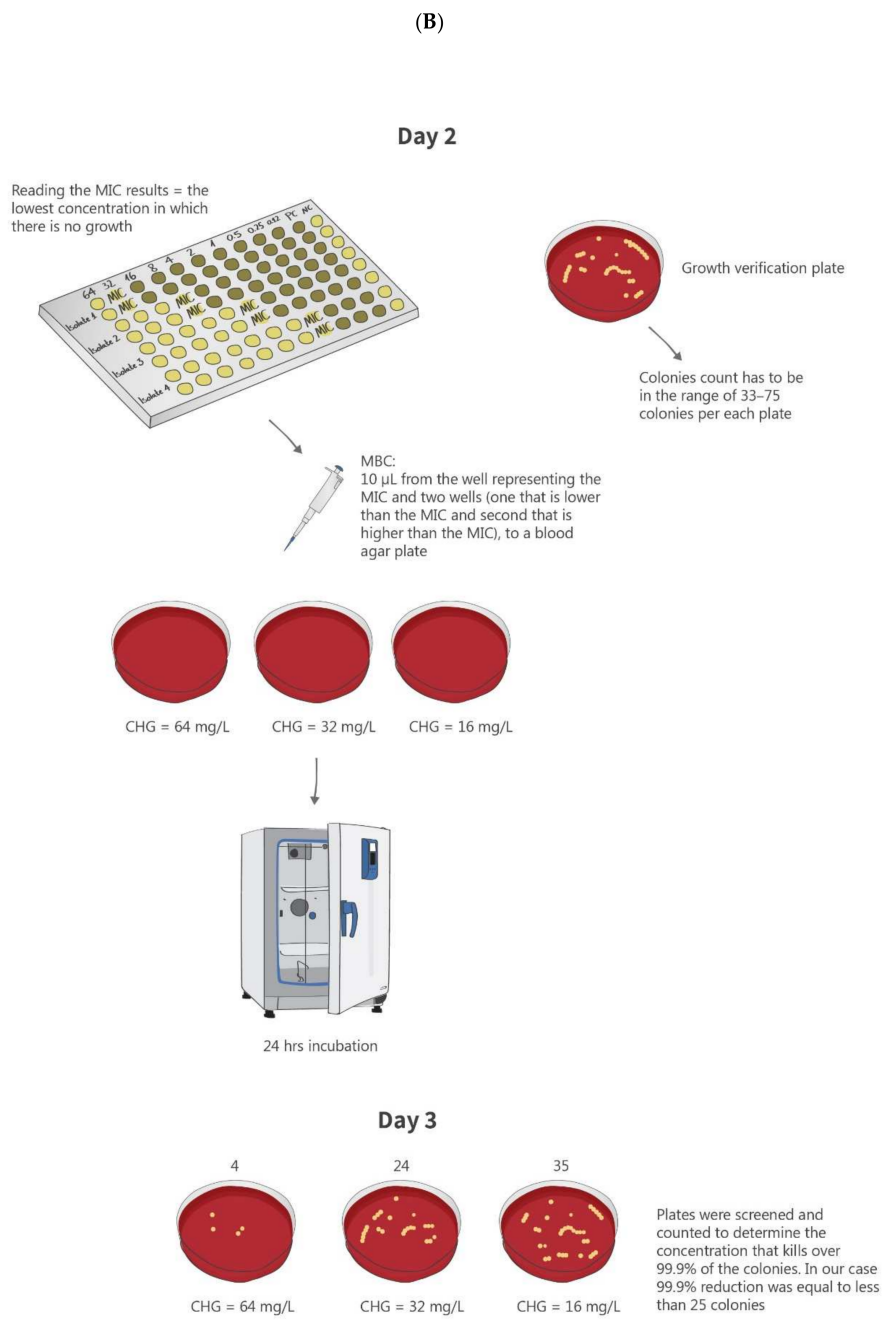
Schematic of the methods used for CHG susceptibility testing. (**A**). Determination of minimum inhibitory concentration (MIC) for CHG. (**B**). Determination of minimum bactericidal concentration (MBC) for CHG.

The MIC was determined using the broth microdilution method as previously described [46] and as recommended by the American Society for Microbiology (ASM) guidelines [47]. Figure 3 summarizes the methods used for CHG susceptibility testing.

##### Bacterial Inoculum

All isolates were seeded on blood agar plates (BD Diagnostics, Sparks, MD, United States) and incubated at 37 °C for 24 h prior to the experiment. Isolated colonies from each culture were suspended in 0.9% saline to create a 0.5 McFarland turbidity, which is equivalent to approximately 1–2 × 10^8^ colony-forming units (CFU)/mL. This stock was then diluted with 0.9% saline to a final concentration of 1–2 × 10^6^ CFU/mL.

Two strains were used for quality control: *S. aureus* ATCC strain 29213, which is recommended by the American Society for Microbiology (ASM) guidelines [47], and the ATCC strain 700699, which has reduced susceptibility to CHG [48].

##### CHG Preparation

Serial dilutions of a primary CHG stock (200 mg/mL; Sigma-Aldrich, Rehovot, Israel) using the Mueller–Hinton broth (MHB) (Hy Laboratories Ltd., Rehovot, Israel) were prepared in order to create 10 final concentrations (0.12, 0.25, 0.5, 1, 2, 4, 8, 16, 32, and 64 µg/mL).

For each CHG concentration and for each isolate, the following positive and negative controls were prepared: the positive control (PC) included the bacterial inoculum in the MHB without CHG and the negative control (NC) included CHG with the saline, without the bacterial inoculum. Each CHG concentration and control was tested in duplicates.

##### Determination of the MIC

The assay was performed in 96-well polypropylene plates (Sigma-Aldrich, Rehovot, Israel). The bacterial inoculum (100 µL) was seeded at the same concentration (final concentration of 5 × 10^6^ CFU/mL) to all wells, aside from the NC wells. A different concentration of CHG was added to each column of wells, except for the PC wells. Quality control strains were added to the dedicated wells. Following seeding, the 96-well plates were incubated at 37 °C for 24 h. Thereafter, controls were checked, and then the 96-well plates were screened for presence of bacterial growth. Growth was indicated by a single turbidity dot. The MIC was defined as the lowest CHG concentration that inhibited bacterial growth. When duplicate wells presented different results, the test was repeated. Reduced susceptibility to CHG was defined according to the epidemiological cut-off of the MIC ≥ 4 (mg/L) [27,45].

#### 4.3.2. Minimum Bactericidal Concentration (MBC) Determination

MBC is the concentration that kills ≥99.9% of cells. In order to determine the MBC as recommended by the ASM guidelines [47], 10 µL samples from the well representing the MIC and two wells treated with the CHG concentrations just below and just above the MIC were transferred to a blood agar plate. The agar plate was incubated at 37 °C for 24 h. The plates were screened for colonies, which were counted to determine the MBC. The MBC was determined as the concentration in which fewer than 25 colonies grew [47].

### 4.4. Genotypic Susceptibility to CHG

Molecular detection of *qac* A/B genes was performed using the real-time polymerase chain reaction (PCR) technique. To this end, DNA was extracted from bacteria using a GenElute^TM^ Bacterial Genomic kit (Sigma-Aldrich, Rehovot, Israel) as per the manufacturer’s instructions. PCR was performed using the primers and a probe as previously described [49] under the following conditions: 50 °C for 2 min, 95 °C for 20 s, and 40 cycles of 95 °C for 35 s, 60 °C for 30 s.

### 4.5. Panton–Valentine Leucocidin (pvl), mecC, and mecA Detection

Presence of the Panton–Valentine leucocidin (*pvl*) gene was detected using an eazyplex^®^ MRSAplus kit (AmplexDiagnostics GmbH, Germany) which is based on the rapid isothermal amplification reaction. This qualitative molecular test detects the presence of the *pvl* toxin gene and identifies MRSA isolates by detection of the *mec*A and *mec*C genes. When the *mec*A, *mec*C, or *pvl* genes are present in the detected *S. aureus* isolate, specific amplification products are generated. Due to the binding of a fluorescence dye to these double-stranded DNA amplification products, presence of the corresponding genes is visualized by real-time fluorescence. The procedure was performed according to the manufacturer’s instructions.

### 4.6. Statistical Analysis

Univariate tests were applied to analyze the differences in the phenotypic and genotypic characteristics and between CHG susceptibility of MSSA isolates vs. MRSA isolates.

Comparisons between groups were made using the chi-squared or non-parametric Wilcoxon–Mann–Whitney rank-sum test for independent samples for the categorical and continuous variables, respectively.

Statistical significance was determined with the *p*-value < 0.05. The data were analyzed using SPSS version 25.

## Figures and Tables

**Table 1:** Characteristics of clinical isolates.

Characteristic	MSSA *n* = 98 *n* (%)	MRSA *n* = 92 *n* (%)	Total Isolates *n* = 190 *n*	*p*-Value ^1^
Sample source				0.865
Blood cultures	34 (50.7)	33 (49.3)	67	
Wounds	64 (52)	59 (48)	123	
Presence of *pvl*				**0.001** ^2^
Yes	11 (28.2)	28 (71.8)	39	
No	87 (57.6)	64 (42.4)	151	
Susceptibility to mupirocin				**<0.001**
Resistant	2 (11.1)	16 (88.9)	18	
Sensitive	96 (59.25)	76 (40.75)	162	
Mupirocin’s MIC (mg/L)				**0.023**
Mean	15.3	178.75	94.45	
Median (Q1, Q3)	0.125 (0.94, 0.19)	0.19 (0.102, 0.865)	0.125 (0.094, 0.25)	

^1^ Chi-squared test, Mann–Whitney test. ^2^ Bold values indicate statistical significance.

**Table 2:** Susceptibility to CHG among clinical isolates.

Characteristic	MSSA *n* = 98 *n* (%)	MRSA *n* = 92 *n* (%)	Total Isolates *n* = 190 *n*	*p*-Value ^1^
Reduced susceptibility to CHG				**<0.001** ^2^
Yes	3 (12)	22 (88)	25	
No	95 (57.6)	70 (42.4)	165	
CHG’s MIC (mg/L)				**<0.001**
Mean	1.62	2.5	2.04	
Median (Q1, Q3)	2 (1, 2)	2 (2, 3.5)	2 (1, 2)	
CHG’s MBC (mg/L)				**<0.035**
Mean	2.23	2.45	2.33	
Median (Q1, Q3)	2 (1, 2)	2 (2, 2)	2 (2, 2)	
*qac* A/B presence				**<0.001**
Yes	4 (16)	21 (84)	25	
No	94 (57)	71 (43)	165	

^1^ Chi-squared test, Mann–Whitney test. ^2^ Bold values indicate statistical significance.

**Table 3:** Clinical and epidemiological characteristics of CHG-tolerant vs. CHG-susceptible isolates.

Characteristic	Reduced Susceptibility to CHG		Total Isolates *n* = 190 *n* (%)	*p*-Value ^1^
	Yes *n* = 25 *n* (%)	No *n* = 165 *n* (%)		
*qac* A/B presence				**<0.0001** ^2^
Yes	10 (40)	15 (60)	25	
No	15 (9)	150 (81)	165	
Methicillin susceptibility				**<0.0001**
Resistant (MRSA)	22 (24)	70 (76)	92	
Sensitive (MSSA)	3 (3)	95 (97)	98	
Mupirocin susceptibility				**0.001**
Resistant	7 (39)	11 (61)	18	
Sensitive	18 (10.5)	154 (89.5)	172	
Mupirocin’s MIC (mg/L)				0.066
Mean	257.44	69.74	94.45	
Median (Q1, Q3)	0.19 (0.125, 256)	0.064 (0.094, 0.25)	0.125 (0.094, 0.25)	
*pvl* presence				**0.548**
Yes	4 (10.25)	35 (89.75)	39	
No	21 (12.3)	130 (87.7)	171	
Sample source				**0.020**
Blood culture	14 (21)	53 (79)	67	
Wounds	11 (8.95)	112 (91.05)	123	

^1^ Chi-squared test, Mann–Whitney test. ^2^ Bold values indicate statistical significance.

**Table 4:** Clinical and epidemiological characteristics of CHG-tolerant isolates vs. CHG-susceptible MSSA and MRSA isolates.

Characteristic	Reduced Susceptibility to CHG among MSSA Isolates (*n* = 98)		*p*-Value ^1^	Reduced Susceptibility to CHG among MRSA Isolates (*n* = 92)		*p*-Value ^1^
	Yes (*n* = 3)	No (*n* = 95)		Yes (*n* = 22)	No (*n* = 70)	
*qac* A/B presence			0.717			**0.004** ^2^
Yes	0	4 (100)		10 (47.6)	11 (52.4)	
No	3 (3.2)	91 (96.8)		12 (16.9)	59 (83.1)	
Mupirocin susceptibility			**<0.0001**			0.161
Resistant	1 (50)	1 (50)		6 (37.5)	10 (62.5)	
Sensitive	2 (2.1)	94 (97.9)		16 (21.1)	60 (78.9)	
Mupirocin’s MIC (mg/L)			0.846			0.243
Mean	85.4	13.1		280.9	146.6	
Median (Q1, Q3)		0.125 (0.094, 0.19)		0.22 (0.125, 776)	0.125 (0.101, 0.25)	
*pvl* presence			0.532			0.152
Yes	0	11(100)		4 (14.3)	24 (85.7)	
No	3 (3.4)	84 (96.6)		18 (28.1)	46 (71.9)	
Sample source			0.960			**0.009**
Blood culture	1 (2.9)	33 (97.1)		13 (39.4)	20 (60.6)	
Wounds	2 (3.1)	62 (96.9)		9 (15.3)	50 (84.7)	

^1^ Chi-squared test, Mann-Whitney test. ^2^ Bold values indicate statistical significance.

## Data Availability

Data are contained within the article.
